# Probe-Type Multi-Core Fiber Optic Sensor for Simultaneous Measurement of Seawater Salinity, Pressure, and Temperature

**DOI:** 10.3390/s24061766

**Published:** 2024-03-08

**Authors:** Chengcheng Feng, Hao Niu, Hongye Wang, Donghui Wang, Liuxia Wei, Tao Ju, Libo Yuan

**Affiliations:** 1 Key Laboratory of In-Fiber Integrated Optics of Ministry of Education, College of Physics and Optoelectronic Engineering, Harbin Engineering University, Harbin 150001, China; cc.feng@hrbeu.edu.cn (C.F.); wanghongye92@hrbeu.edu.cn (H.W.); wangdonghui1994@hrbeu.edu.cn (D.W.); 2 School of Optoelectronic Engineering, Guilin University of Electronic Technology, Guilin 541004, China; haoniu@mails.guet.edu.cn (H.N.); weiliuxia@guet.edu.cn (L.W.); lbyuan@guet.edu.cn (L.Y.)

**Keywords:** multi-core optical fiber sensor, surface plasmon resonance, Fabry–Pérot interference, fiber Bragg grating, multiple parameters sensing

## Abstract

In this article, we propose and demonstrate a probe-type multi-core fiber (MCF) sensor for the multi-parameter measurement of seawater. The sensor comprises an MCF and two capillary optical fibers (COFs) with distinct inner diameters, in which a 45° symmetric core reflection (SCR) structure and a step-like inner diameter capillary (SIDC) structure filled with polydimethylsiloxane (PDMS) are fabricated at the fiber end. The sensor is equipped with three channels for different measurements. The surface plasmon resonance (SPR) channel (CH_SPR_) based on the side-polished MCF is utilized for salinity measurement. The fiber end air cavity, forming the Fabry–Pérot interference (FPI) channel (CH_FPI_), is utilized for pressure and temperature measurement. Additionally, the fiber Bragg grating (FBG) channel (CH_FBG_), which is inscribed in the central core, serves as temperature compensation for the measurement results. By combining three sensing principles with space division multiplexing (SDM) technology, the sensor overcomes the common challenges faced by multi-parameter sensors, such as channel crosstalk and signal demodulation difficulties. The experimental results indicate that the sensor has sensitivities of 0.36 nm/‰, −10.62 nm/MPa, and −0.19 nm/°C for salinity, pressure, and temperature, respectively. As a highly integrated and easily demodulated probe-type optical fiber sensor, it can serve as a valuable reference for the development of multi-parameter fiber optic sensors.

## 1. Introduction

The ocean covers more than 70% of the Earth’s surface, influencing the global environment and climate while supporting human survival and development. Therefore, as a prerequisite for understanding ocean ecology, studying the ocean environment, and exploiting ocean resources, ocean exploration is of great significance to human society [1,2]. Modern ocean exploration technology primarily utilizes sound, light, electricity, and magnetic detection platforms to sense and analyze the physical, chemical, and biological parameters of the ocean [3]. These technologies include space remote sensing, ship-borne observation, ocean buoys, submersibles, and other advanced techniques. Recently, the ocean detection network technology composed of multi-functional small sensors has become the focus of development because of its unique advantages of wide detection range, low cost, and real-time monitoring [4,5].

Optical fiber sensors are emerging as an innovative sensing technology, gradually replacing traditional electronic sensors, and are recognized as a crucial component of ocean networking detection technology [6]. These sensors offer several advantages, such as multifunctionality, miniaturization, resistance to electromagnetic interference, easy waterproofing, and integration into optical fiber communication networks. In ocean research, numerous parameters require detection, among which salinity, temperature, and pressure are indispensable parameters for the study of ocean physics, as they enable researchers to calculate key factors such as ocean density, depth, and dynamics to achieve real-time analysis of ocean currents, tides, and stratification. Consequently, optical fiber sensors predominantly find application in the measurement of seawater salinity, temperature, and pressure for ocean exploration purposes [7,8]. Numerous institutions and scholars have conducted research on optical fiber sensors for the measurement of salinity, temperature, and pressure [9,10,11,12,13,14,15,16]. For instance, L. Ji et al. [17] introduced a π phase-shifted FBG sensor enclosed in a metal thin-walled cylinder, enabling the extensive range and high-resolution measurement of seawater pressure. D. Xue et al. [18] combined microelectromechanical systems (MEMSs) with FPI interferometers to propose a highly sensitive fiber optic sensor that can simultaneously measure seawater salinity and temperature. Similarly, Y. Liu et al. [19] utilized tapered polarization-maintaining fiber and FBG to design a fiber optic sensor based on the Sagnac loop for the simultaneous measurement of seawater salinity and temperature. Numerous research studies have explored optical fiber sensors for measuring ocean parameters. However, only a limited number of sensors have been able to simultaneously measure salinity, temperature, and pressure because incorporating additional measurement channels significantly complicates the sensor design, fabrication, and signal demodulation processes. Y. Zhao et al. [20] proposed a three-channel optical fiber sensor based on the SPR effect, which can measure seawater salinity, pressure, and temperature concurrently by employing different sensitive films. Nonetheless, the process of coating multiple sensitizing materials at the end of the fiber is extremely challenging. Moreover, accommodating three SPR dips within a limited spectral range increases the risk of spectral overlap. J. Liu et al. [21] developed a micro-nano fiber-based three-channel sensor using the MZI technique to simultaneously measure the temperature, salinity, and pressure of seawater. However, the mechanical strength of the micro-nano fiber is notably low, posing a high risk of fracture during actual measurements. Furthermore, utilizing a single measurement mechanism to obtain three-parameter measurements introduces the challenge of sensitivity crosstalk, complicating signal demodulation. A variety of sensing mechanisms have been successfully applied to fiber optic sensors for seawater measurements, the most commonly used mechanisms being SPR, FPI, and FGB. SPR-sensing structures are particularly useful for seawater salinity measurements due to their high refractive index (RI) sensitivity, simple construction, and low cost. Fiber-ended FP sensing structures are frequently utilized for pressure measurements because of their high sensitivity and the absence of additional mechanical sensitizing structures. FBG is an excellent choice for temperature measurement or compensation of sensors. Compared to using a single sensing mechanism for multi-parameter measurements, utilizing different measurement mechanisms can effectively prevent channel crosstalk and spectral overlap and simplify signal demodulation. However, integrating multiple sensing mechanisms on a single optical fiber is challenging and requires innovative structural design and complex preparation processes. To address these challenges, G. An et al. [22] designed a fiber optic sensor capable of measuring all three parameters by integrating three different sensing mechanisms—namely SPR, FBG, and FPI. A tilted fiber Bragg grating (TFBG) was used to achieve the excitation of the SPR effect. The SPR transmission spectrum was then reflected to the input fiber by a chirped fiber Bragg grating (CFBG), effectively solving the problem of incompatibility between the transmitted SPR-sensing structure and the reflected fiber end FP sensing structure. However, TFBG-SPR typically has a low sensitivity and requires a complex polarization control optical path.

Complex structural design and difficult signal demodulation have been the main problems hindering the development and application of multi-parameter fiber optic sensors, while multi-core fiber optic sensors provide an effective idea to solve these problems. In this article, we propose and demonstrate a probe-type optical fiber sensor that allows for multi-parameter measurement in seawater. The sensor is composed of an MCF and COFs and integrates three measurement channels. The CH_SPR_ based on the side-polished MCF is utilized to measure salinity. The CH_FPI_ relies on an air cavity to measure pressure and temperature. The CH_FBG_ inscribed in the central core of the MCF serves as a temperature compensation component. To enhance the performance of the sensor, we incorporate two distinct microstructures. The first is the SIDC structure, which streamlines the process of filling the PDMS and enhances the repeatability of sensor preparation. The second is the SCR structure, which effectively directs the transmission spectrum of SPR into the symmetric side core of the MCF, solves the compatibility problem between the SPR-sensing structure and fiber end FP sensing structure, and realizes the probe-type structure design. The sensor employs three distinct sensing principles in its three channels, which, combined with SDM technology, enables independent demodulation of the measurement results for each channel. This approach effectively prevents crosstalk between the channels and significantly reduces the complexity of demodulating multi-parameter measurement results. The sensor has demonstrated promising performance in experimental tests, with sensitivity values of 0.36 nm/‰ for salinity (range: 0 to 60‰), −10.62 nm/MPa for pressure (range: 0.1 to 0.5 Mpa), and −0.19 nm/°C for temperature (range: 25 to 85 °C). As a highly integrated and easily demodulated probe-type optical fiber sensor, it has exciting application prospects in the multi-parameter measurement of shallow seawater, tidal estuaries, and saltwater lakes.

## 2. Operating Principle and Fabrication

**Figure 1:**
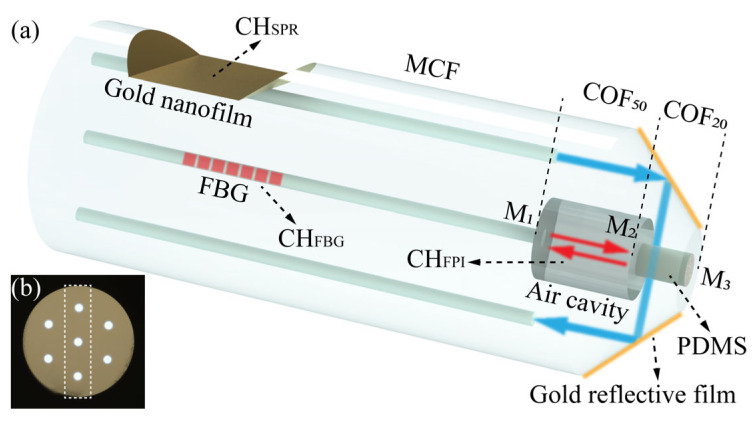
(**a**) Schematic diagram of the proposed probe-type fiber optic sensor. (**b**) Cross-view micrograph of the seven-core fiber.

A schematic diagram of the probe-type optical fiber sensor for the measurement of seawater salinity, pressure, and temperature is shown in Figure 1a; the sensor consists of an MCF, a COF with an inner diameter of 50 μm (COF_50_), and a COF with an inner diameter of 20 μm (COF_20_). The COF_50_ and COF_20_ have lengths of approximately 50 and 30 μm, respectively. The interior of the COF_50_ comprises air, while the interior of the COF_20_ is filled with PDMS. These components, along with the MCF central core, create an air cavity to generate FPI. Changes in temperature and pressure cause the PDMS to deform, thus affecting the size of the air cavity. Consequently, the CH_FPI_ based on the air cavity can be utilized to measure the temperature and pressure of seawater. The MCF is side-polished until one side core is exposed, and the polished surface is then plated with a 50 nm thick gold film to create a CH_SPR_ to measure seawater salinity. The fiber end is shaped into a 45° cone frustum using micro-grinding technology and coated with a gold reflective film on the grinding surface, resulting in an SCR structure. The SPR transmission spectrum, which carries information about the sample salinity, is reflected twice by the structure, entering the symmetric side core, and subsequently received by the spectrometer. Furthermore, the FBG inscribed in the central core of the MCF is referred to as CH_FBG_ and is employed to compensate for the temperature crosstalk of the CH_SPR_ and the CH_FPI_. The MCF used by the sensor is a seven-core fiber with a core diameter of 9 μm and a cladding diameter of 125 μm. The side core is located 37.5 μm from the central core. As depicted in Figure 1b, only the three cores enclosed within the dotted line frame are utilized in this study.

The change in seawater salinity causes a simultaneous change in its RI. Therefore, the CH_SPR_, which is highly sensitive to the change in the RI, can be employed to measure seawater salinity. The theoretical model of the side-polished MCF SPR is based on the Kretschmann configuration [23], which has been extensively discussed in our previous work [24]. The transmission spectrum of the CH_SPR_ can be calculated by the following formula:

(1) T(λ)=exp(−4πλIm(neff)L)

where *n_eff_* represents the effective RI of the side-polished fiber, *λ* denotes the wavelength of light transmitted in the core, and *L* corresponds to the SPR-sensing region, which measures approximately 10 mm in this study. The light transmitted in the fiber core is a broad-spectrum light, and the SPR effect induces a resonance dip in the transmission spectrum, with the lowest point of the dip referred to as the resonance wavelength. Changes in seawater salinity (Δ*S*) cause shifts in the resonant wavelength, so the salinity measurement sensitivity of the CH_SPR_ can be expressed as follows:

(2) $$S_{s}=\frac{\Delta S}{\Delta \lambda_{r}}$$

Pressure and temperature measurements are performed using the CH_FPI_. According to Fresnel’s law, the sensor consists of three reflector surfaces: M_1_, located at the interface between the central core and air; M_2_, situated at the interface between air and PDMS; and M_3_, positioned at the interface between PDMS and seawater, as depicted in Figure 1. The reflectance values of M_1_ and M_2_ remain unaffected by seawater salinity, measuring 3.60% and 2.85%, respectively. However, the reflectance of M_3_ is only 0.06% at a seawater salinity of 30‰. Given that M_1_ and M_2_ have similar reflectance characteristics and M_3_ exhibits an extremely low reflectance, the FPI primarily occurs within the air cavity of the COF_50_. Accordingly, the resonance wavelength of the FPI can be determined using the following formula:

(3) $$\lambda_{m}=\frac{4\pi nL}{\left(2m+1\right)\pi }$$

where *n* represents the RI of the medium inside the FP cavity, *L* denotes the length of the cavity, and *m* corresponds to the interference order.

Further, under the disturbance of seawater pressure (Δ*P*) and temperature (Δ*T*), the shift of the *m*-order resonance wavelength can be expressed as follows:

(4) $$\Delta \lambda_{P}=\frac{4}{2m+1}(L\frac{\partial n}{\partial P}+n\frac{\partial L}{\partial P})\Delta P$$

(5) $$\Delta \lambda_{T}=\frac{4}{2m+1}(L\frac{\partial n}{\partial T}+n\frac{\partial L}{\partial T})\Delta T$$

**Figure 2:**
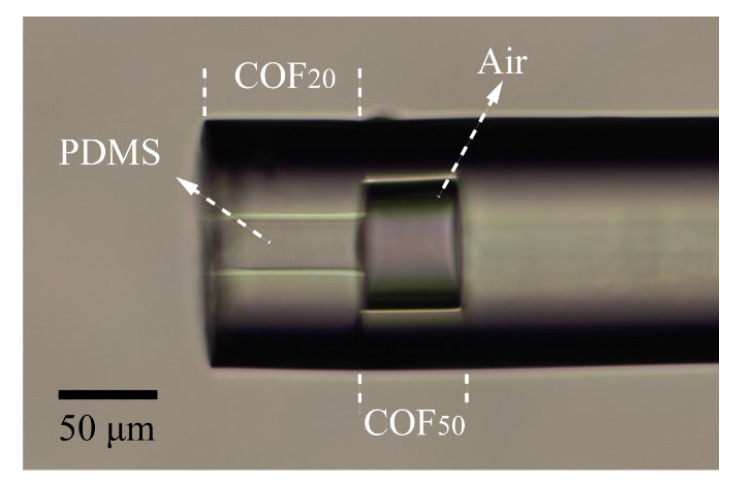
Micrograph of the SIDC structure filled with PDMS.

Elastic polymers such as PDMS and ultraviolet glue are commonly used materials for FPI-based optical fiber pressure sensors. They change the *L* or *n* of the FP cavity through elastic deformation when subjected to pressure. There are two typical pressure-sensing structures: a cavity filled with polymer [25,26] or a cavity with a polymer film placed at the end [27,28]. The former structure is easier to prepare as the liquid polymer completely fills the cavity due to the capillary effect. However, it results in a poor contrast of the FPI spectrum due to the polymer and fiber interface having extremely low reflectivity. On the other hand, the FPI spectrum based on the latter structure offers suitable contrast but is challenging to prepare due to the precise operations required for filling the polymer at the picolitre level. To achieve a stable and controllable PDMS filling process and obtain a high spectral contrast, we propose a novel structure called the SIDC structure. This structure is created by fusing the COF_50_ and COF_20_. Figure 2 illustrates the SIDC structure filled with PDMS. In this structure, the strength of the capillary effect is inversely proportional to the inside diameter of the capillary. As a result, PDMS only exists within the COF_20_ region, while the COF_50_ remains an air-filled cavity. The thickness of the filled PDMS film is determined by the length of the COF_20_, while the length of the FP air cavity is determined by the length of the COF_50_. This allows for precise control over the dimensions and properties of the PDMS and air cavity sections within the sensor structure.

**Figure 3:**
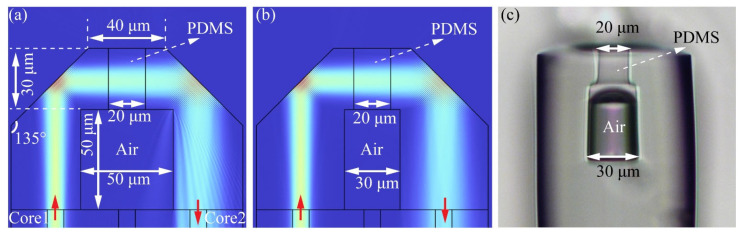
Simulation results of the SCR structure based on (**a**) the COF_50_ and (**b**) the COF_30_. (**c**) Micrograph of the PDMS-filled SCR structure based on the COF_30_.

Previous studies have established the viability of the symmetric core reflection structure [29], but further investigation is necessary to determine the impact of the SIDC structure on reflection efficiency. Based on the actual preparation technique, we initially established the length of the COF_20_ for the PDMS filling to be 30 μm. Within our laboratory, we have two types of COFs suitable for creating the FP air cavity, and their inner diameters are 50 and 30 μm (COF_30_). Therefore, we first discuss the effect of the capillary inner diameter on the reflection efficiency of the SIDC structure. Figure 3a,b depict the simulation results of the reflectance of the SIDC structure based on the COF_50_ and COF_30_, respectively, at an incident wavelength of 635 nm. The two simulated structures differ solely in the inner diameter of the COF, while the other structural parameters and filling media remain constant. It is evident that the presence of PDMS in the COF_20_ does not significantly affect the beam propagation, and the COF_50_ only slightly impacts the propagation of the divergent beam. The reflection efficiency of the proposed structure is determined by the ratio of the output power of core 1 to the input power of core 2. The reflection efficiencies of the structures depicted in Figure 3a,b were 61.02% and 61.25%, respectively, with no significant difference between them. In the subsequent PDMS filling test, the COF_30_ is filled with PDMS, as illustrated in Figure 3c. This occurred because the capillary effect of the COF_30_ is similar to that of the COF_20_. As a result, our final approach is a combination of the COF_50_ and COF_20_.

Considering the typical length range of the FP cavity, which is usually between 50 and 100 μm, we also investigated the effect of the COF_50_ length on the reflectance. Simulation results indicate that with COF_50_ lengths of 50, 75, and 100 μm, the corresponding reflectance values of the structure were 61.02%, 54.76%, and 46.16%, respectively. Hence, it can be concluded that shorter structures exhibit higher reflectivity.

**Figure 4:**
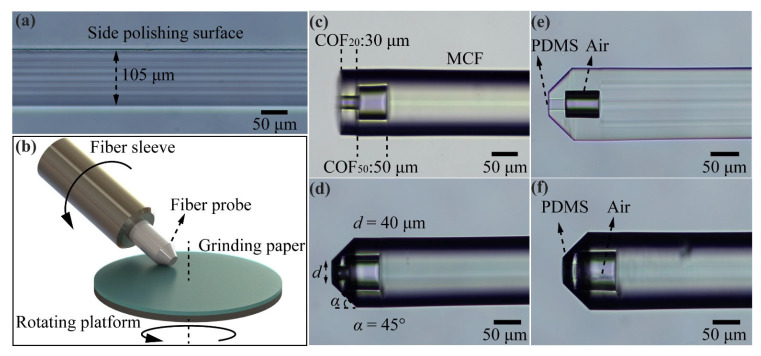
(**a**) Side-view micrograph of the polished MCF. (**b**) Schematic diagram of the optical fiber end micro-grinding system. (**c**) Micrograph of the prepared SIDC structure. (**d**) Fiber end micrograph with the 45° cone frustum structure. (**e**) Micrograph of the fiber end immersed in the PDMS droplet. (**f**) Fiber end micrograph after PDMS curing.

**Figure 5:**
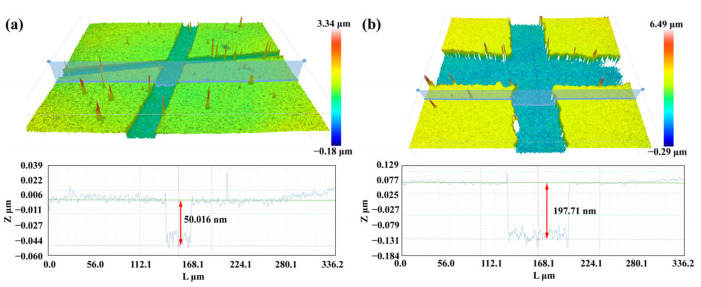
Measurement results of the thickness of the gold film plated on the (**a**) side-polishing surface and (**b**) fiber end.

The fabrication process of the optical fiber sensor involves six steps: FBG inscribing, fiber side-polishing, COF fusion and cutting, fiber end micro-grinding, PDMS filling, and gold film deposition. In the first step of FBG inscribing, a phase mask method is employed utilizing a 248 nm KrF excimer laser. The FBG inscribing system includes a CCD for observing the core, which ensures that the FBG is accurately inscribed in the central core of the MCF. This step is crucial for the precise positioning of the FBG within the fiber core [30,31]. In the next step, the MCF is side-polished using the wheel side-polishing method, which includes a pair of fiber rotators and a pair of CCDs to adjust the core position and observe the remaining thickness of the fiber to ensure that the side core is just exposed. The microscopic image of the side-polished MCF is shown in Figure 4a. In the third step, the MCF and COF_50_ are fused together with cladding alignment using an optical fiber fusion splicer (NT-600s, Notian, Nanjing, China). After fusion, the COF_50_ is cut to a fixed length with a cutting device. This cutting device allows for clear observation of the fusion point between the MCF and COF_50_, helping to accurately adjust and control the cutting position to achieve the desired length of the remaining COF_50_. Following this, the COF_20_ is fused to the front end of the COF_50_ using the same method. Figure 4c illustrates the MCF-COF_50_-COF_20_ sample that was prepared, providing a visual representation of the completed structure. In the subsequent step, the 45° cone frustum structure is created using the fiber end micro-grinding method, as depicted in Figure 4b. The prepared sample is secured in a fiber sleeve and brought into contact with the grinding paper. Two motors are utilized to drive both the fiber sleeve and the rotating platform, enabling the grinding process of the fiber end. Figure 4d shows the MCF-COF_50_-COF_20_ sample after undergoing the grinding process. The diameter of the remaining fiber end, denoted as *d*, is directly influenced by the duration of the grinding process. Furthermore, the cone angle, represented as *α*, is determined by the angle formed between the fiber and the rotating platform. In the fifth step, the prepared sample is immersed in a PDMS droplet. Due to the capillary effect, the PDMS gradually fills the capillary structure, flowing along the channel created by the COF_20_. The filling process continues until it reaches the interface between the COF_20_ and COF_50_, as depicted in Figure 4e. At this point, the PDMS stops advancing further, resulting in a PDMS-filled capillary structure that is confined within the designated region. In the subsequent step, any excess PDMS remaining at the end of the fiber is carefully wiped off. The sample is then cured at a temperature of 75 °C. Figure 4f displays a microscopic image of the cured sample. Then, a gold reflection film with a thickness of approximately 200 nm is deposited on the fiber end surface, and a gold film with a thickness of approximately 50 nm is deposited onto the side-polishing surface by an ion sputtering apparatus (ETD-900M, Elaborate, Beijing, China). The thickness of the gold films is measured by a three-dimensional surface morphology analyzer (S neox-90, Sensofar, Barcelona, Spain). The measured thicknesses are represented in Figure 5a for the side-polishing surface and Figure 5b for the fiber end surface. Finally, the gold film on the top surface of the 45° cone frustum structure is gently wiped with grinding paper to prevent the transmitted light from being reflected to the central core, thus improving the signal-to-noise ratio of the FPI and FBG reflection spectra.

To establish a connection between the proposed probe-type optical fiber sensor and the light source and detector, a fan-in-fan-out device (FIFO) is utilized. This FIFO device, based on SDM technology, facilitates the efficient coupling of light signals between the external components and any core of the MCF.

To measure the reflectance of the SIDC structure, a 635 nm semiconductor laser and a silicon-based optical power detector (PM121D, Thorlabs, Newton, NJ, USA) are used. The measured reflectance, found to be 35%, was lower than the simulation result. Several factors contributed to this discrepancy. Firstly, the symmetry of the structure could affect the reflectance, as any deviation from perfect symmetry may result in reduced reflectivity. Secondly, the roughness of the fiber end grinding surface can also impact the reflectance, as it may introduce the scattering and absorption of light. Lastly, the insertion loss of the FIFO can cause a reduction in the overall signal strength, thus affecting the measured reflectance.

## 3. Experimental Setup

**Figure 6:**
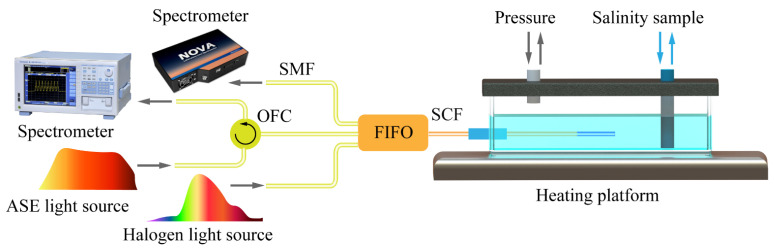
Experimental setup of the proposed probe-type sensor.

The sensing system based on the proposed sensor is schematically shown in Figure 6. The sensor was securely sealed within a pressure chamber and fully immersed in the salinity sample. To regulate the temperature within the chamber, a heating platform was positioned at the bottom. The pressure was controlled by a connected pressure pump, while the salinity was adjusted using an injection pump. Halogen light (360–2500 nm, HL2000, Ideaoptics, Shanghai, China) entered the side core by the FIFO, inducing the SPR effect. The SPR transmission spectrum containing the salinity information was reflected into the symmetric side core at the end of the fiber and received by the spectrometer (325–1100 nm, NOVA, Ideaoptics, Shanghai, China) after passing through the FIFO once again. On the other hand, amplified spontaneous emission (ASE) light within the range of 1530–1600 nm was directed into the central core of the MCF through the optical fiber circulator (OFC) and FIFO. The reflection spectra of FPI and FBG containing pressure and temperature information were received by the spectrometer (600–1700 nm, AQ6370C, Yokogawa, Tokyo, Japan) after passing through the FIFO and OFC once again.

For the salinity measurement, the samples were sodium chloride aqueous solution with a concentration of 0‰ to 60‰, the pressure in the chamber was 0.1 Mpa, and the temperature was room temperature. For the pressure measurement, the sensor was immersed in a sample with a salinity of 30‰, the temperature in the chamber was room temperature, and the pressure was gradually increased from 0.1 to 0.5 Mpa. For temperature measurement, the sensor was also immersed in a sample with a salinity of 30‰, the pressure was maintained at 0.1 Mpa, and the temperature was gradually increased from 25 °C to 85 °C.

It is important to mention that our sensing system currently requires two sets of light sources and spectrometers due to the limited detection range of our current spectrometers. This limitation results in increased costs and complexity in operating the system. However, this issue can be effectively addressed by implementing a spectrometer with an expanded detection range, such as the AQ6374 spectrometer (350–1750 nm, Yokogawa, Tokyo, Japan).

## 4. Results and Discussion

### 4.1. Salinity-Sensing Characteristic

Salinity sensing was realized by the CH_SPR_ of the sensor. Since the SPR effect could not occur at the interface between the gold film and air, the spectrum collected by the CH_SPR_ in air was set as the reference spectrum, and the ratio between the spectrum collected in the salinity sample and the reference spectrum was the SPR spectrum related to salinity.

**Figure 7:**
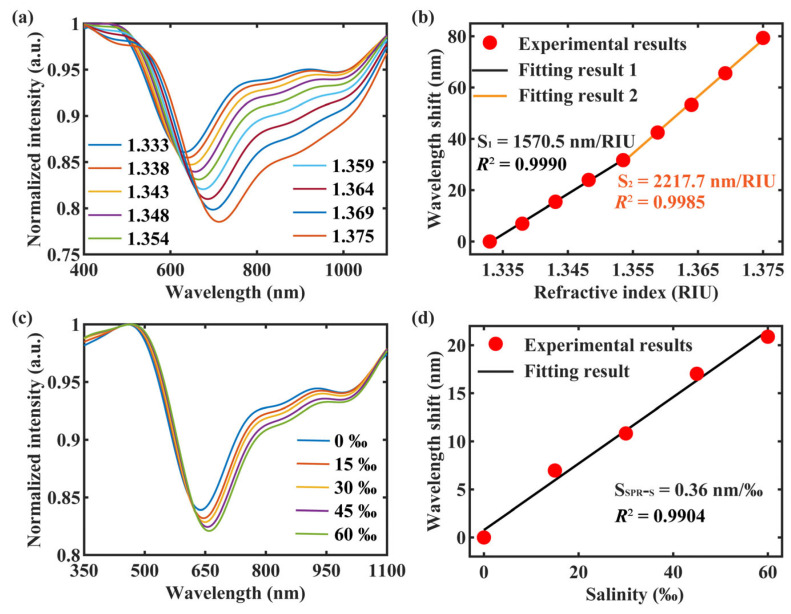
(**a**) Response of the CH_SPR_ to RI. (**b**) The variation of Δλ_SPR_ with RI and the corresponding fitting results. (**c**) Response of the CH_SPR_ to salinity. (**d**) The variation of Δλ_SPR_ with salinity and the corresponding fitting results.

Firstly, we demonstrated the sensing characteristics of the CH_SPR_ using the RI samples, and the experimental results are shown in Figure 7a. With the RI increase, the SPR spectrum gradually shifted toward longer wavelengths, indicating a redshift. The corresponding shift of the resonance wavelength with varying salinity is presented in Figure 7b. As the RI increased from 1.333 to 1.375 RIU, the resonance wavelength of the CH_SPR_ showed a redshift of 79.35 nm.

A piecewise linear fitting method was employed to analyze the experimental results. In the RI range of 1.333 to 1.354 RIU, the experimental data aligned well with a linear fit, having an R-squared value of 0.9990 and a sensitivity of 1570.5 nm/RIU. Similarly, for the salinity range of 1.354 to 1.375 RIU, the experimental data also fit a linear trend, with an R-squared value of 0.9985 and a sensitivity of 2217.7 nm/RIU.

Then, the salinity-sensing characteristics of the CH_SPR_ were tested. Given that seawater averages a salinity of 35‰, we set the concentration of our salinity samples ranging from 0‰ to 60‰. The experimental results, displayed in Figure 7c, clearly illustrate the gradual redshift of the SPR spectrum with increasing sample salinity. Additionally, Figure 7d presents the corresponding shift in resonance wavelength with varying salinity. As the salinity increased from 0‰ to 60‰, the resonance wavelength showed a redshift of 20.88 nm, which aligned with a linear fitting (R^2^ = 0.9904), indicating a salinity measurement sensitivity of 0.36 nm/‰.

The FP air cavity and the FBG inscribed in the central core were not influenced by changes in sample salinity. Therefore, the CH_FPI_ and CH_FBG_ of the sensor did not exhibit a response to variations in salinity.

### 4.2. Pressure-Sensing Characteristic

**Figure 8:**
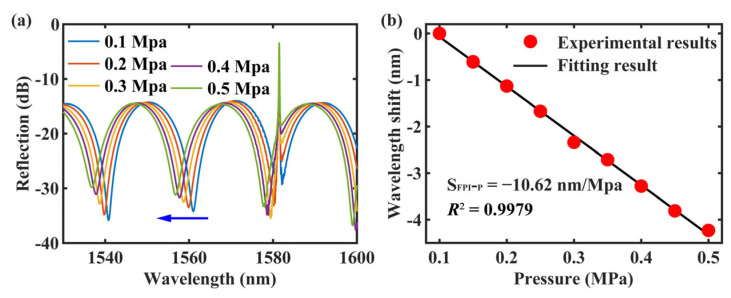
(**a**) Response of the CH_FPI_ and CH_FBG_ to pressure (the blue arrow indicates a blueshift). (**b**) The variation of Δλ_FPI_ with pressure and the corresponding fitting results.

The CH_FPI_ of the sensor was designed to measure pressure by utilizing the deformation of PDMS under pressure. This deformation causes a change in the *L* of the FP cavity, enabling pressure sensing. Figure 8a displays the spectrum of the central core output, including the reflection spectra of the CH_FPI_ and CH_FBG_, when the pressure in the chamber was altered. It is important to note that the FPI and FBG spectra are independent of each other.

As the pressure increased, the resonance dip of the FPI spectrum gradually shifted toward shorter wavelengths, indicating a blueshift. However, the peak of the FBG spectrum remained unchanged. Figure 8b visualizes the shift in the FPI resonance wavelength (Δλ_FPI_) with varying pressure. As the pressure increased from 0.1 to 0.5 MPa, the resonance wavelength showed a blueshift of 4.23 nm. A linear fitting analysis of these data revealed a correlation with an R^2^ value of 0.9979 and a sensitivity of −10.62 nm/MPa.

The pressure-sensing structure’s sensitivity and measurement range are closely related to the filled material. PDMS has a low Young’s modulus of 5.11 Mpa, which provides a suitable sensitivity but limits the measurement range. To extend the pressure measurement range of the sensing structure, filled materials with higher Young’s modulus, such as photosensitive resins, can be used. Conversely, using a material with a lower Young’s modulus, such as gelatin [32], can significantly enhance sensitivity but will result in a smaller measurement range. Therefore, the appropriate filled material can be chosen to meet different application scenarios for this pressure-sensing structure. For example, the current experimental results support the proposed sensor being applied to scenarios where the seawater depth is within 40 m. The sensor characteristics need to be re-tested and calibrated after replacing the filling material.

It is widely acknowledged that the RI of a medium is often correlated with pressure. However, in this study, a pressure change of 0.4 MPa was found to have a minimal effect on the RI of the air within the FP cavity, the RI of the salinity sample, and the effective RI of the fiber. Consequently, no discernible changes were observed in the SPR and FBG spectra during the pressure measurement experiments.

In the practical application of deep-sea measurement, it is crucial to consider the impact of high pressure on salinity and the complexity of sensitivity crosstalk between the CH_SPR_ and CH_FPI_. Researchers have extensively investigated the effects of seawater salinity, temperature, and pressure on the RI, as discussed in reference [33]. The findings from this reference indicate that with every 10 m increase in seawater depth, the pressure of seawater rises by 0.101 MPa, resulting in a corresponding RI increase of 1.49 × 10^−4^ RIU. The resolution of the spectrometer used to collect the SPR signal was *σ* = 1.97 nm, and the RI sensitivity of the CH_SPR_ channel was *S*_1_ = 1570.5 nm/RIU. As a result, the RI resolution was calculated as *R*_RI_ = *σ*/*S*_1_ = 1.3 × 10^−4^ RIU. This implies that obtaining an accurate relationship between *λ*_SPR_ and pressure necessitates a pressure change step of at least 8.49 MPa (equivalent to a depth step of 840 m). However, due to the limitations of our self-made pressure chamber, we were constrained to a pressure test range of 0.1–0.5 MPa. These limitations were imposed by the experimental conditions at hand.

### 4.3. Temperature-Sensing Characteristic

The PDMS also experienced deformations in response to changes in temperature, which consequently affected the *L* of the FP cavity. As a result, temperature sensing was achieved through the CH_FPI_ of the sensor. Additionally, since the composite refractive index of the gold film was dependent on temperature, the measurement results of the CH_SPR_ were also influenced by temperature variations. To account for this, the CH_FBG_ was employed as a temperature compensation unit.

**Figure 9:**
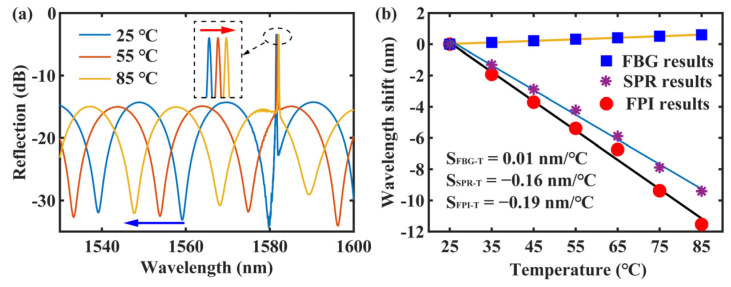
(**a**) Response of the CH_FPI_ and CH_FBG_ to temperature (the blue arrow represents a blueshift and the red arrow represents a redshift). (**b**) The variations of Δλ_FBG_, Δλ_SPR_, and Δλ_FPI_ with temperature and their corresponding fitting results (color lines).

Figure 9a illustrates the reflectance spectra of the FPI and FBG as the temperature changed. With increasing temperature, the FPI dip gradually blueshifted, while the FBG peak gradually redshifted. Figure 9b displays the shifts of the FBG peak (Δλ_FBG_), Δλ_SPR_, and Δλ_FPI_ in correlation with temperature. Notably, all three shifts conformed to linear fits, exhibiting sensitivities of 0.01, −0.16, and −0.19 nm/°C, respectively.

### 4.4. Simultaneous Measurement of Three Parameters

The sensitivity matrix is a commonly employed approach for correcting the results of multi-parameter measurements. Based on the experimental findings, Δλ_SPR_ was associated with variations in Δ*S* and Δ*T*, Δλ_FPI_ was linked to Δ*P* and Δ*T*, while Δλ_FBG_ solely related to Δ*T*. Consequently, the measurement results of the sensor for these three parameters can be mathematically expressed as follows:

(6) $$\left[\begin{array}{l} \Delta \lambda_{SPR}\\ \Delta \lambda_{FP}\\ \Delta \lambda_{FBG} \end{array}\right]=\left[\begin{array}{ccc} 0.36 & 0 & -0.16\\ 0 & -10.62 & -0.19\\ 0 & 0 & 0.01 \end{array}\right]\left[\begin{array}{c} \Delta S\\ \Delta P\\ \Delta T \end{array}\right]$$

Further, the sensitivity matrix employed to correct the measurement results of the sensor is as follows:

(7) $$\left[\begin{array}{c} \Delta S\\ \Delta P\\ \Delta T \end{array}\right]=\left[\begin{array}{ccc} 2.78 & 0 & 44.44\\ 0 & -0.09 & -1.79\\ 0 & 0 & 100 \end{array}\right]\left[\begin{array}{l} \Delta \lambda_{SPR}\\ \Delta \lambda_{FP}\\ \Delta \lambda_{FBG} \end{array}\right]$$

**Figure 10:**
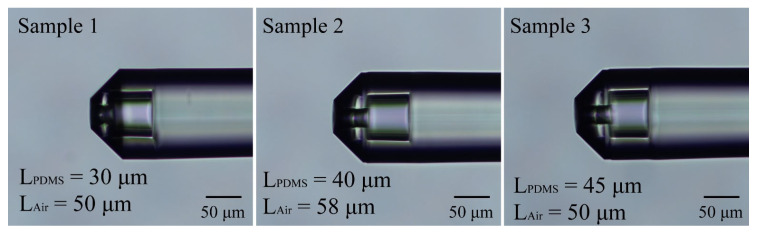
The micrographs of the fiber end of the three sensors prepared in this work.

In this experiment, three sensors with similar structures were prepared. Due to the precision of the fiber fixed-length cutting device, there were slight variations in the filling lengths of the PDMS (L_PDMS_) and the lengths of the FP air cavities (L_Air_) among the sensors, as depicted in Figure 10. The sensing characteristics of sample 1 were demonstrated in detail, and a performance comparison of all three sensors is presented in Table 1. It is apparent from the results that the pressure sensitivity decreased, and the temperature sensitivity increased with a longer length of L_PDMS_. This result can be attributed to the amount of PDMS filled in the sensors. To improve the consistency of sensor preparation and performance, it is crucial to enhance the accuracy of the fiber fixed-length cutting device.

Table 2 provides a performance comparison between the proposed sensor and previous optical fiber seawater sensors. It is evident that most fiber optic sensors are of the transmission type or can only measure two parameters of seawater. Reference [20] introduced a probe-type fiber sensor based on the SPR effect, allowing for the three-parameter measurement of seawater. However, its reflection spectrum consists of three SPR dips, which have the risk of crosstalk. Similarly, the three-parameter measurement scheme proposed in reference [21] also has a serious crosstalk problem, which greatly increases the difficulty of signal demodulation. On the other hand, reference [22] proposed a probe-type fiber optic seawater sensor that utilizes three different sensing principles to address the issue of crosstalk. However, its measurement system includes a reference FP cavity, which compromises the stability of the system. The presence of the reference FP cavity can be influenced by environmental disturbances, thereby affecting the measurement results of the underwater probe. In comparison, the proposed sensor offers notable advantages in terms of its compact structure, measurement sensitivity, and system stability. It has a flexible probe structure and overcomes the limitations posed by channel crosstalk, resulting in improved performance and reliability.

## 5. Conclusions

In this article, we presented a probe-type fiber optic sensor designed specifically for measuring the salinity, temperature, and pressure of seawater. The sensor comprised three different types of fiber: MCF, COF_50_, and COF_20_. At the fiber end, a SIDC structure was incorporated, along with an SCR structure filled with PDMS. The sensor featured three individual sensing channels: CH_SPR_, which was employed for salinity measurement, CH_FPI_ for pressure measurement, and CH_FBG_ for temperature compensation. Integrated with SDM technology, the proposed sensor enabled independent demodulation of the measurement results from each channel. This solves the challenges related to channel crosstalk and the complicated demodulation of multi-parameter signals. The sensor is characterized by its flexible probe structure, high repeatability in fabrication, low channel crosstalk, and ease of signal demodulation. Based on current experimental results, the sensor has significant potential for multi-parameter measurement in shallow seawater, tidal estuaries, and saltwater lakes. In the future, the proposed sensor has two directions of development. The first direction is to add multiple SPR-sensing channels based on side-polished side cores for the detection of heavy metals and biological parameters in the ocean, which is a unique advantage of the MCF sensor. The second direction is to enhance the measurement capability for the deep ocean, such as high pressure, which is an important factor for the practical application of ocean exploration.

## Figures and Tables

**Table 1:** Performance comparison of three sensors prepared in this work.

Sensor Sample	1	2	3
*S*_S_ (nm/‰)	0.36	0.33	0.34
*S*_P_ (nm/MPa)	−10.62	−9.53	−9.16
*S*_T_ (nm/°C)	−0.19	−0.21	−0.23

**Table 2:** Comparison of optical fiber sensors for seawater measurement.

Technique	Type	*S*_S_ (nm/‰)	*S*_P_ (nm/MPa)	*S*_T_ (nm/°C)	Reference
SPRs	Transmission	0.30	/	−2.40	[9] (2023)
SPR, MMI	Transmission	0.36	−1.42	/	[10] (2021)
Sagnac loop	Transmission	0.36	/	0.62	[19] (2024)
SPRs	Reflective	0.56	2.84	−1.80	[20] (2019)
MZIs	Transmission	0.63	3.78	−2.31	[21] (2022)
FPI, CFBG, SPR	Reflective	0.12	11.69	0.01	[22] (2023)
SPR, FPI, FBG	Reflective	0.36	−10.62	−0.19	This work

## Data Availability

Data are contained within the article.
